# Automated identification of elemental ions in macromolecular crystal structures

**DOI:** 10.1107/S1399004714001308

**Published:** 2014-03-20

**Authors:** Nathaniel Echols, Nader Morshed, Pavel V. Afonine, Airlie J. McCoy, Mitchell D. Miller, Randy J. Read, Jane S. Richardson, Thomas C. Terwilliger, Paul D. Adams

**Affiliations:** a Physical Biosciences Division, Lawrence Berkeley National Laboratory, Berkeley, CA 94720-8235, USA; b Department of Haematology, University of Cambridge, Cambridge Institute for Medical Research, Wellcome Trust/MRC Building, Cambridge CB2 0XY, England; c Stanford Synchrotron Radiation Lightsource, SLAC National Accelerator Laboratory, Menlo Park, CA 94025, USA; d Joint Center for Structural Genomics, http://www.jcsg.org, USA; e Department of Biochemistry, Duke University Medical Center, Durham, NC 27710, USA; f Los Alamos National Laboratory, Los Alamos, NM 87545-0001, USA; g Department of Bioengineering, University of California at Berkeley, Berkeley, CA 94720-1762, USA

**Keywords:** refinement, ions, *PHENIX*

## Abstract

Many macromolecular model-building and refinement programs can automatically place solvent atoms in electron density at moderate-to-high resolution. This process frequently builds water molecules in place of elemental ions, the identification of which must be performed manually. The solvent-picking algorithms in *phenix.refine* have been extended to build common ions based on an analysis of the chemical environment as well as physical properties such as occupancy, *B* factor and anomalous scattering. The method is most effective for heavier elements such as calcium and zinc, for which a majority of sites can be placed with few false positives in a diverse test set of structures. At atomic resolution, it is observed that it can also be possible to identify tightly bound sodium and magnesium ions. A number of challenges that contribute to the difficulty of completely automating the process of structure completion are discussed.

## Abstract 2

The solvent-picking procedure in *phenix.refine* has been extended and combined with *Phaser* anomalous substructure completion and analysis of coordination geometry to identify and place elemental ions.

## 1. Introduction   

**Figure 1:**
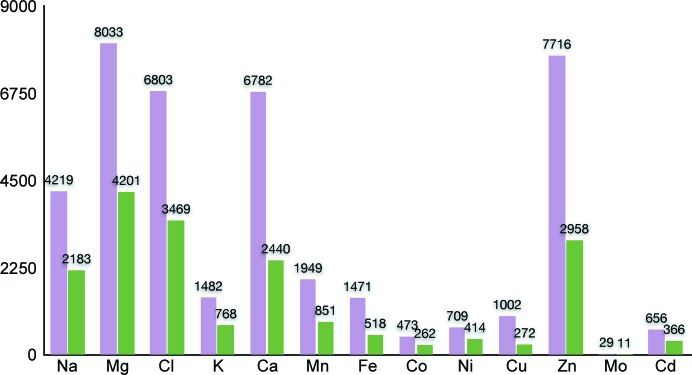
Frequency of elemental ion types in X-ray crystal structures in the PDB, as of September 2013. This does not include instances of these elements as part of other molecules (*e.g.* heme, chlorophyll or iron–sulfur clusters), but both oxidation states of iron, copper and molybdenum are counted here. Pink bars represent the counts of all deposited structures containing the specified ions; green bars are for structures filtered at 90% sequence identity.

In addition to organic molecules, macromolecular crystals frequently contain ordered monoatomic ions. These ions often account for a nontrivial amount of the scattering density in the unit cell and are often physiologically relevant, aiding in catalysis and substrate binding as well as stabilizing protein folds (Glusker, 1991 ▶; Harding *et al.*, 2010 ▶). They are also common components in many crystallization solutions, often at high concentrations. Statistics for some of the most common elemental ions in the Protein Data Bank (PDB; Bernstein *et al.*, 1977 ▶; Berman *et al.*, 2000 ▶) are shown in Fig. 1 ▶. Automated structure determination and analysis of metal-binding proteins or nucleic acids depends on reliable building of these sites, a task that is complicated by the similar chemical and scattering properties of different ions.

Correct determination of the elemental identity requires detailed knowledge of the binding characteristics of each candidate metal. Much information has been compiled on this subject (Harding *et al.*, 2010 ▶); however, although several tools have been described for predicting or validating suspected ions (Nayal & DiCera, 1996 ▶; Hooft *et al.*, 1996 ▶; Zheng *et al.*, 2008 ▶), the lack of automated tools incorporating this knowledge currently requires the individual crystallographer to place ions manually. As a result, there are numerous examples of undetected ions in published crystal structures and, in some cases, incorrectly assigned elements. Examining the residual difference map alone does not always yield an unambiguous conclusion, as incorrect ions can still be fitted to the density through refinement of their atomic displacement parameters and occupancies to compensate for the difference in scattering. Identification of the lighter elements such as sodium, magnesium or chlorine is particularly problematic, especially when they bind nonphysiologically or when the structure determination is at low resolution.

Previous work has identified rules and metrics to aid in automatically characterizing ionic species. Linus Pauling’s second rule, the electrostatic valence rule (Pauling, 1929 ▶), has been used to calculate bond-valence parameters that quantitatively relate bond lengths and bond valences (Brown & Altermatt, 1985 ▶; Brese & O’Keeffe, 1991 ▶). These parameters have then been used, with moderate success, to automatically characterize unknown ions by screening for reasonable valence values (Nayal & Di Cera, 1994 ▶, 1996 ▶). Additional improvements on the method have included examining the balance of bond valences around the ion to help improve specificity (Müller *et al.*, 2003 ▶). In parallel, Harding and others have systematically characterized the general patterns in the chemical environment of different ions by examining both small-molecule structures in the Cambridge Structural Database (Allen, 2002 ▶) and protein structures in the PDB (Glusker, 1991 ▶; Harding, 1999 ▶, 2000 ▶, 2001 ▶, 2002 ▶, 2004 ▶, 2006 ▶; Rulísek & Vondrásek, 1998 ▶; Dokmanić *et al.*, 2008 ▶; Zheng *et al.*, 2008 ▶). A purely physical approach based on anomalous scattering may also be used to identify heavier elements (Mueller-Dieckmann *et al.*, 2007 ▶; Thorn & Sheldrick, 2011 ▶), even in cases where the chemical environment is insufficient to distinguish ions from water.

Here, we describe a procedure that combines these methods, using the chemical environment, electron density and anomalous scattering data, when available, to identify and refine the most common monoatomic cations (Na, Mg, K, Ca, Mn, Fe, Co, Cu, Ni, Zn and Cd) in high-resolution X-ray crystal structures. A majority of ‘native’ (*i.e.* physiologically relevant) zinc and calcium binding sites in a diverse test set can be placed automatically, with few false positives. When candidates cannot be differentiated, a list of viable options is presented for manual inspection. Our method is implemented as part of the *PHENIX* software for automated macromolecular crystallography (Adams *et al.*, 2010 ▶).

## 2. Methods   

### 2.1. Flagging incorrectly modeled waters   

The core routine of the method runs by iterating, in parallel, over all of the water molecules in the structure that have been previously placed and refined and classifying them based on scattering properties and other indicators. (Already built ions are not modified, although post-refinement validation is performed to flag suspicious assignments.) An incorrectly assigned water is considered likely to be a ‘heavier’ ion (for example, calcium or a transition metal) if it meets one of several criteria after refinement, including an unusually low isotropic *B* factor (*B*_iso_), a residual peak in the likelihood-weighted *mF*_o_ − *DF*_c_ difference map (where *m* and *D* are calculated as described in Read, 1986 ▶; Lunin & Skovoroda, 1995 ▶; Urzhumtsev *et al.*, 1996 ▶), high occupancy (above 100%) or detectable anomalous signal (if available). The cutoffs for these analyses are all user-adjustable options, but we have empirically chosen as defaults a minimum *B*_iso_ cutoff of 1.0 Å^2^, peak cutoffs of 3.0σ for the *mF*_o_ − *DF*_c_ and anomalous maps and *f*′′  above 0 (calculated by *Phaser* as described in §[Sec sec2.4]2.4). Waters that may still be incorrectly assigned that fail these tests but have a significantly lower isotropic *B* factor or higher 2*mF*_o_ − *DF*_c_ map value compared with the mean for all water atoms are considered as potential ‘light’ ions (sodium or magnesium). We exclude from consideration any waters with a negative difference map peak (below −3.0σ) or weak 2*mF*_o_ − *DF*_c_ density (empirically chosen as below 1.8σ), as these are considered to be unreliable.

Two additional environmental filters are used to select designated ‘waters’ for further analysis. The presence of nearby phosphate O atoms from a nucleotide (*e.g.* ATP or GTP) will also flag the putative water as a possible coordinating atom (Mg, Mn or Ca). We also take into account unusually close contacts with other O atoms: based on the criteria used by *Probe* (Word *et al.*, 1999 ▶; Chen *et al.*, 2010 ▶), a cutoff of 2.4 Å for oxygen–oxygen distances is used here.

### 2.2. Filtering candidate elements   

Once a site has been identified as being potentially incorrectly modeled as water, the list of candidate ions is filtered based on the chemical and electron-density characteristics of the site. The default search candidates, selected based on frequency in the PDB, are magnesium, calcium, zinc and chloride, but a list of elements to search for can also be provided. The current library includes parameters for sodium, magnesium, chloride, potassium, calcium, manganese, iron, cobalt, copper, nickel, zinc and cadmium. For most purposes the procedure is more effective when the elements under consideration are explicitly specified, since the constraints can be relaxed if the identity of the bound ion candidates is known in advance. The parameters used are outlined in Supplementary Tables S1 and S2^1^, based on Rulísek & Vondrásek (1998 ▶), Harding (2000 ▶, 2001 ▶), Muller *et al.* (2003 ▶), Dokmanić *et al.* (2008 ▶) and Zheng *et al.* (2008 ▶).

**Figure 2:**
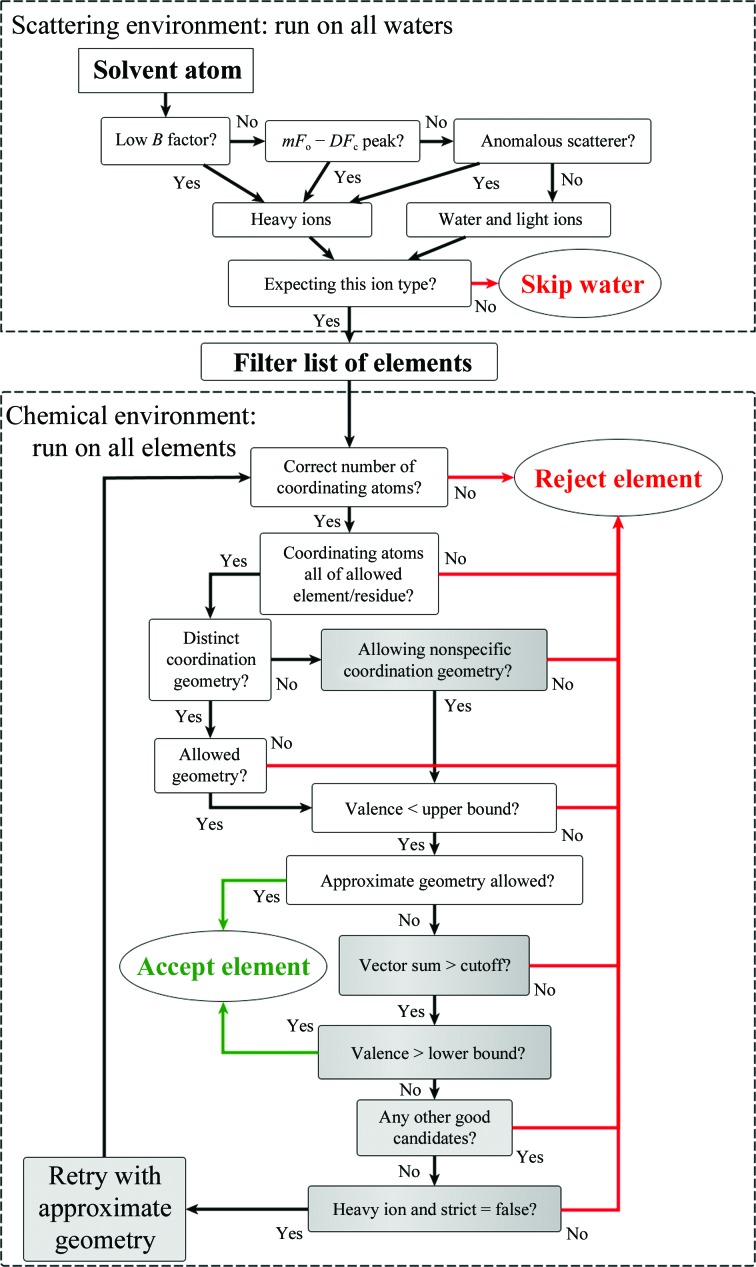
Schematic of the decision tree used for ion identification in *phenix.refine*. Operations shaded in gray are required for identification of light ions, but may be waived for heavy ions if no suitable elements are identified using all criteria.

To filter the candidates, the properties of the coordinating atoms (within 3.5 Å of the site) are examined first, taking crystal symmetry into account. For common motifs such as carboxyl or phosphate groups, where the close proximity of carbon or phosphorus might otherwise exclude a candidate element, the bond connectivity is taken into account when identifying close contacts. A decision tree for narrowing the list of possible elements was derived based on these different properties (Fig. 2 ▶). In cases of lower resolution or incomplete models, where coordination shells are often partial, certain parameters such as bond valence may be given less emphasis when other evidence such as anomalous scattering is available and the user has not specified the strict use of geometry tests. Additionally, while it is possible to specify the ion identities to screen for, likely alternatives are presented when a flagged site does not pass either the strict or weak filters for any of the candidates. Possible halide atoms are identified on the basis of their coordination by positively charged amide groups and cations.

### 2.3. Chemical properties   

The analysis of chemical environment takes into account several different factors, including not only the identity of the coordinating atoms, but also the coordination geometry, total number of coordinating atoms and residue-type preferences (Rulísek & Vondrásek, 1998 ▶; Dokmanić *et al.*, 2008 ▶; Zheng *et al.*, 2008 ▶). For example, only Co, Fe, Ni, Zn or Cu are allowed to be coordinated by the sulfur in cysteine, while methionine coordination is restricted to Co, Ni and Cu. For magnesium, strict octahedral coordination is required; other ions have looser rules, although unfavorable geometry may be used to exclude candidates. To avoid making an erroneous assignment owing to model errors, coordinating atoms are excluded if not fully supported by the electron density.

#### 2.3.1. Bond valence and VECSUM   

The bond-valence sum is an estimate of the total charge of an ion based on the distances between it and its contacts. To calculate the bond-valence sum (BVS) for a given ion identity, we used the bond-valence parameters tabulated by Brown & Altermatt (1985 ▶) and Brese & O’Keeffe (1991 ▶). Using the equation taken from Müller *et al.* (2003 ▶) (1, 2) [Disp-formula fd2], the total sum was calculated from the distances of the coordinating atoms. Here, *r_ij_* is the bond-valence parameter for the ion and a coordinating atom, *d_ij_* is the distance between them and *p_j_* is the percentage occupancy of the ion, 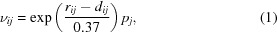


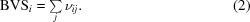



Similarly, the vector sum was calculated by summing vectors (3) to each coordinating atom, with magnitudes equal to the valence contribution of that atom. Here, *r_ij_* is a unit vector pointing from the ion to the coordinating atom,
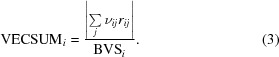



For a more detailed discussion on the background and methodology behind the BVS calculations, see Brown (2009 ▶).

### 2.4. Anomalous scattering   

The occupancy, *B* factor and *mF*_o_ − *DF*_c_ residual peak height are all used to determine whether the correct ion identity is likely to be isoelectronic to the currently modeled atom or whether it should include more or fewer electrons. When anomalous data are available, several additional analyses may be used to identify heavier elements. By default, the substructure completion in *Phaser* (McCoy *et al.*, 2007 ▶) is used to place purely anomalous scatterers, which provides an estimate of *f*′′ for each site identified in this way. The *f*′′ values are compared against the expected value for the X-ray wavelength (if known). This aids greatly both in narrowing down the search field and verifying built ions. In addition, the log-likelihood gradient map (de La Fortelle & Bricogne, 1997 ▶; McCoy & Read, 2010 ▶) may optionally be used in analysis of solvent atoms, or alternatively, the less sensitive (but faster) unweighted anomalous residual map (where amplitudes are calculated by subtracting the calculated from the observed anomalous differences). The simple anomalous difference map may also be used, but this has been found to be significantly less effective when a mixture of strong and weak anomalous scatterers is present (Roach, 2003 ▶).

### 2.5. Integration with refinement   

In *phenix.refine* (Afonine *et al.*, 2012 ▶), ion identification is performed directly after water placement with modified settings (a minimum *B* factor of 0 and run every macro-cycle after the first). If anomalous data are available, *Phaser* is used in the first cycle to locate the anomalous scatterers, which are retained for reuse in future cycles. The method then loops over all water molecules, and performs a comprehensive analysis for any meeting the criteria described in §[Sec sec2.1]2.1. When a single suitable ion type is determined for a water molecule, the atom type is converted internally; the occupancy is also reset to 1.0 (or the equivalent fraction for sites on crystallographic special positions) and the isotropic *B* factor is set to the mean for solvent atoms. Both occupancies and (if appropriate) anomalous scattering coefficients are refined for newly placed ions. *B* factors will be refined as anisotropic if the resolution is better than 1.5 Å, or if the resolution is worse than 2.5 Å and the atomic number is at least 19 (potassium).

Because the procedure depends on the accurate placement of isolated single atoms, it is generally not suitable for low-resolution structures. We have found that the water picking performs poorly at resolutions worse than 2.8 Å, and our tests have been restricted to structures with data extending to at least 2.6 Å resolution. While many of the scattering criteria used are equally valid at low resolution, model errors tend to make the analysis of geometry unreliable.

### 2.6. Testing   

**Figure 3:**
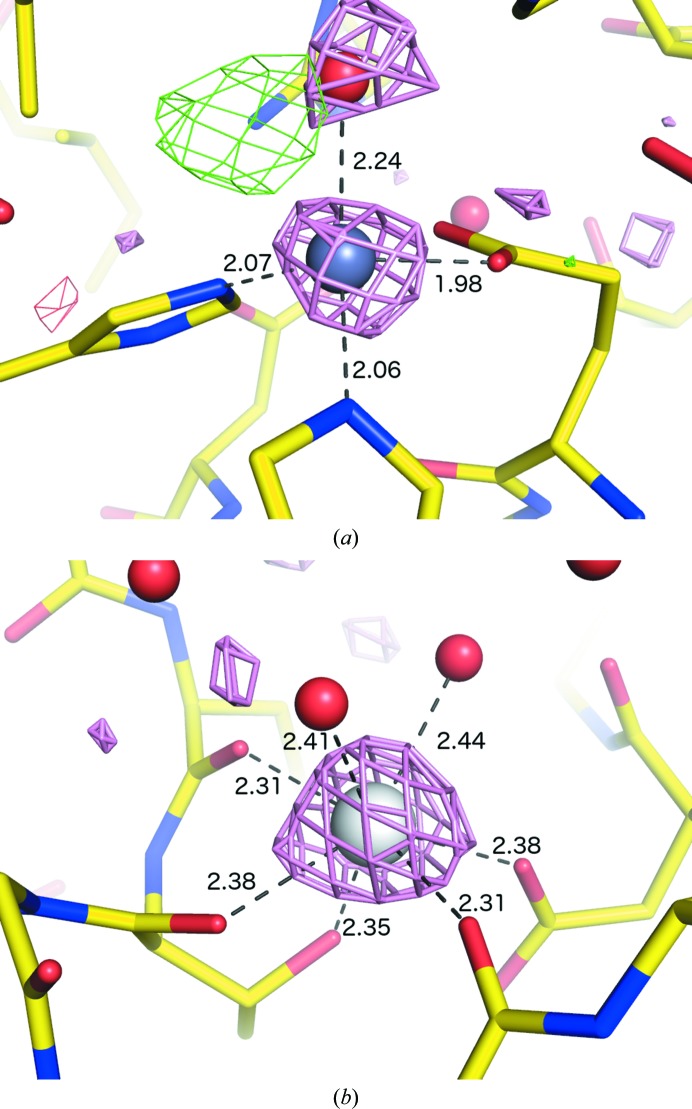
Examples of ion binding in thermolysin (PDB entry 2whz; B. A. Lund, I. Leiros & H.-K.S. Leiros, unpublished work), showing the criteria used to determine the identity of the indicated zinc (*a*) and calcium (*b*) sites starting from refined water molecules. Green and red meshes are *mF*_o_ − *DF*_c_ density at ±3.0σ and pink mesh is anomalous difference density at 3.0σ. Red spheres are water molecules. Distances are labeled in Å.

**Figure 4:**
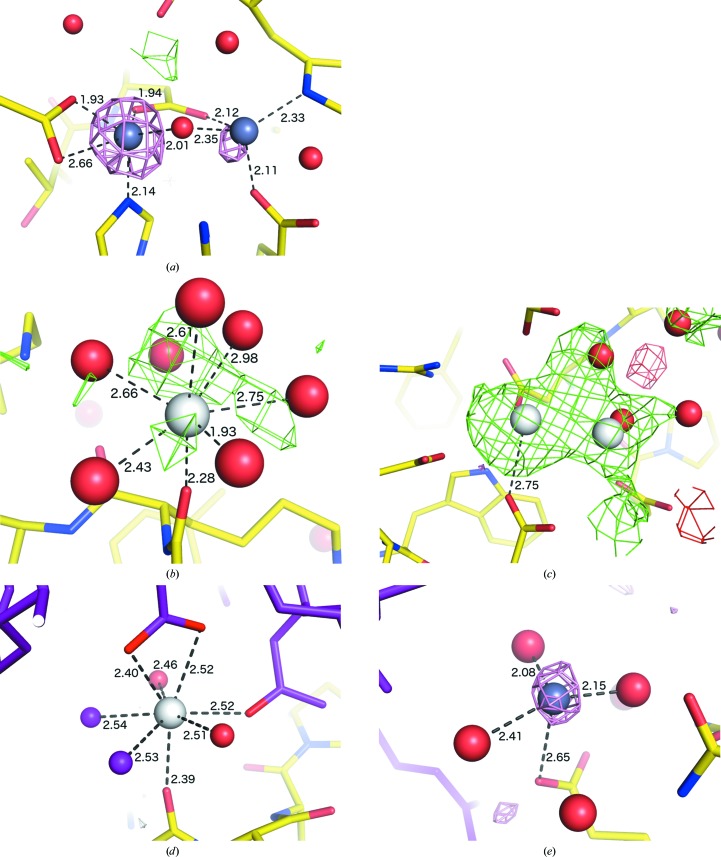
Examples of ion placement using the JCSG data, illustrating potential pitfalls. The models are taken from the PDB, but with maps calculated after attempting to replace the ions in *phenix.refine*. Colors are as in Fig. 3 ▶; anomalous maps are shown in all cases, but signal may be below the contour level. Where shown, purple sticks represent a symmetry-related molecule. Sites shown in (*a*), (*b*) and (*e*) were placed successfully; for (*b*) and (*c*) the originally built atoms are shown for clarity. (*a*) Native Zn sites in 3lub. (*b*) Nonspecific Ca sites in 3lub built and refined as waters by *phenix.refine*. (*c*) Ambiguous sites in 4ecg originally built as Ca^2+^. (*d*) New Ca^2+^ site in 3cjy. (*e*) New Zn^2+^ site in 3h50 (Axelrod *et al.*, 2010 ▶).

**Figure 5:**
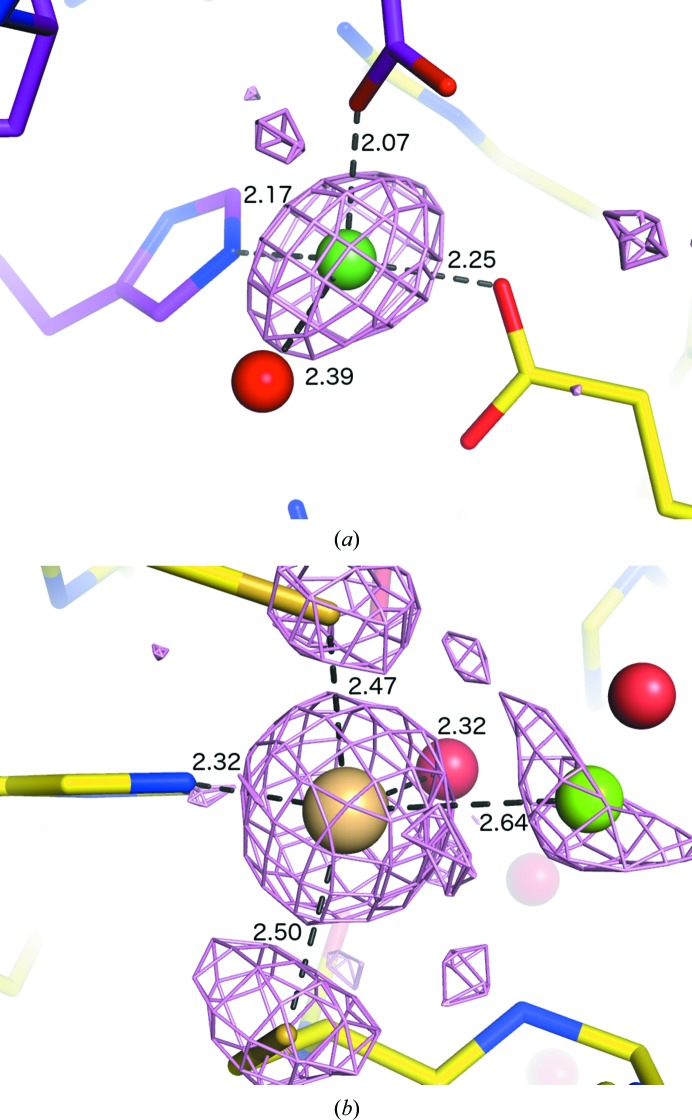
Examples of ion placement by *phenix.refine* for non-zinc transition metals. (*a*) Nickel-binding site in the lattice contacts of 3n6q (Totir *et al.*, 2012 ▶); purple sticks represent a symmetry-related monomer. (*b*) Cadmium (tan) and chloride (green) ions in 3bob (Xu *et al.*, 2008 ▶).

**Figure 6:**
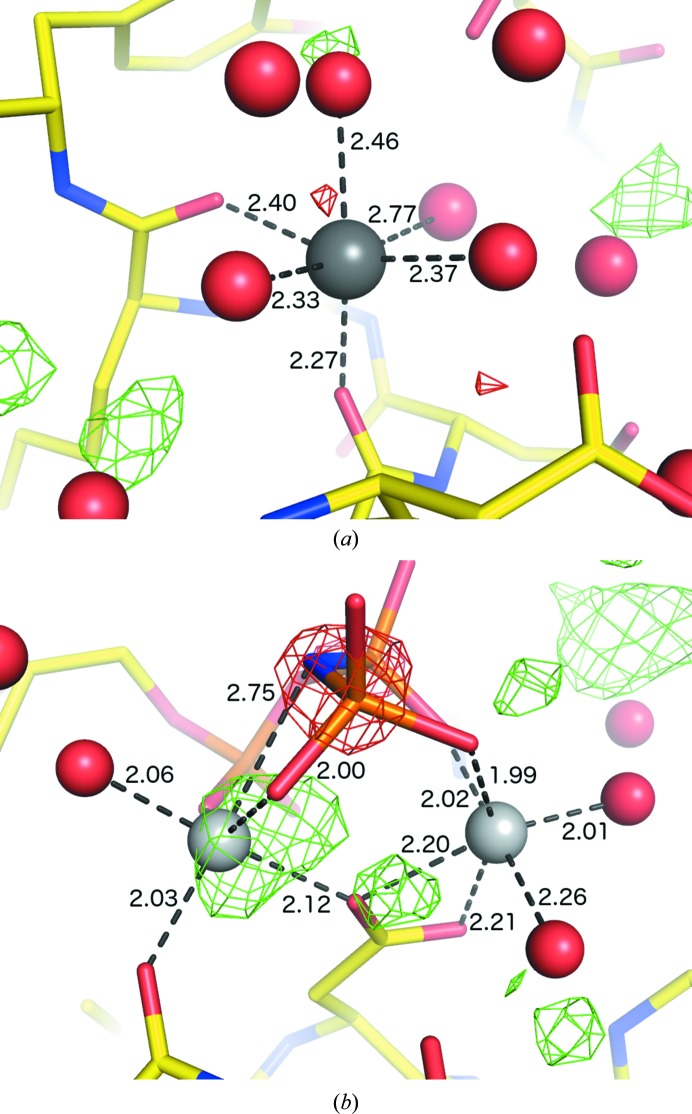
Examples of light-cation placement by *phenix.refine*. (*a*) Sodium binding in thrombin, showing the native site in PDB entry 3si3 (Biela *et al.*, 2012 ▶). (*b*) Magnesium ions in 4dfx (Bastidas *et al.*, 2012 ▶).

For evaluating the performance of the method, we chose test cases consisting of protein crystal structures with anomalous data available (with the exception of calmodulin) and that were solved at resolutions of at least 2.6 Å. The refinement protocol used consists of six macro-cycles of reciprocal-space coordinate, *B* factor, occupancy and anomalous group refinement. Anisotropic *B* factors were refined for all atoms at resolutions greater than 1.2 Å, or all non-water atoms between 1.2 and 1.5 Å resolution; at lower resolutions TLS parameters were refined. At resolutions worse than 1.75 Å we also used automatic optimization of the refinement target weights (Afonine *et al.*, 2011 ▶) and torsion-angle NCS restraints (if applicable; Headd *et al.*, 2014 ▶). The wavelength and expected ions were explicitly specified. All results were visually inspected in *Coot* (Emsley *et al.*, 2010 ▶); Figs. 3–7 were generated in *PyMOL* v.1.2.

## 3. Results   

### 3.1. Calcium and zinc-bound structures   

#### 3.1.1. Calmodulin (Ca^2+^)   

The small regulatory protein calmodulin binds four Ca^2+^ ions, which act as a switch for calmodulin binding to other proteins; we selected the highest resolution structure in the PDB (PDB entry 1exr; Wilson & Brunger, 2000 ▶). Although anomalous data are not available, the resolution (1.0 Å) and quality of the data are high enough that the positions of atoms coordinating native calcium-binding sites are very accurately determined, and therefore also the bond valences. Our method placed three out of four native sites as well as an additional surface site also present in the published model. The missed site was not identified owing to a bond valence that was slightly higher than the cutoff used (approximately 2.3 *versus* the expected 2.0).

#### 3.1.2. Thermolysin (Ca^2+^ and Zn^2+^)   

The protease thermolysin is a popular model system for protein crystallography as it easily forms well diffracting crystals and is commercially available. It contains a catalytic zinc site and four structural calcium sites, all of which bind at full occupancy. We used PDB entry 2whz (B. A. Lund, I. Leiros & H.-K.S. Leiros, unpublished work) owing to its relatively high resolution (1.75 Å, but the Wilson *B* factor and average refined *B* factors suggest inherent diffraction to higher resolution) and the deposition of anomalous data. Because the data are of high quality, our method was able to place all five ions whether or not anomalous data were used, with or without *Phaser* substructure completion (Fig. 3 ▶). However, thermolysin also presents some challenges in the form of static disorder at the Zn^2+^ binding site (Holland *et al.*, 1995 ▶; Thorn & Sheldrick, 2011 ▶), which has been confirmed by multi-wavelength data collection (PDB entry 3fgd; P. Pfeffer, G. Neudert, L. Englert, T. Ritschel, B. Baum & G. Klebe, unpublished work). Because the secondary Zn site is adjacent to the primary site and does not have a recognizable coordination shell, it is instead assigned as a chloride ion when the default element list is used (at this wavelength, Zn and Cl cannot be distinguished by anomalous scattering).

#### 3.1.3. A large-scale benchmark   

As a quantitative and unbiased test of our method, we attempted to identify the Ca^2+^ and Zn^2+^ ions in a set of 54 structures solved by the Joint Center for Structural Genomics (Lesley *et al.*, 2002 ▶) with resolutions ranging from 1.06 to 2.4 Å and anomalous data included in the PDB depositions (Supplementary Table S3). We did not perform any curation of the test set beyond discarding one structure where MTZ file conversion did not work correctly (PDB entry 3pfe), one where the assignment of Zn was confirmed as erroneous (PDB entry 3obc) and three in which the chemical interactions strongly indicated that the wrong element was assigned (PDB entries 3kst, 3l2n and 3rza, which contain calcium coordinated by a histidine side chain). Because a separate set of structures was used for developing and optimizing the protocol, this test was performed ‘blind’, *i.e.* without adjusting the method to improve the success rate.

Results are shown in Table 1 ▶; the overall success rate (defined as the number of ions found by *phenix.refine* that match those in the deposited structure) was 38% for calcium and 79% for zinc. The false-positive rate was extremely low, with only two spurious Ca atoms built in PDB entries 3dzz and 3m83 (Levisson *et al.*, 2012 ▶). We attribute the difference in effectiveness to two reasons: firstly, the stronger anomalous signal for zinc at the traditional synchrotron wavelengths (approximately 1 Å) used for these structures; and secondly, the tendency of zinc sites to be native/structural and bound more tightly, whereas a relatively large fraction of calcium ions are crystallization artifacts owing to the use of calcium salts in solution and are only bound at partial occupancy, usually at the surface of the protein. The method was significantly more successful in replacing the high-occupancy calcium ions (Supplementary Fig. S1), whereas the performance on zinc was independent of occupancy. A similar trend was observed for isotropic *B* factors (Supplementary Fig. S2). A representative example is PDB entry 3lub, which is deposited with 12 Ca^2+^ and 24 Zn^2+^ ions; our method identifies two and 20, respectively. The zinc sites (Fig. 4 ▶ *a*) are internal to the protein and are recognizable by their high 2*mF*_o_ − *DF*_c_ and anomalous map levels. In contrast, the calcium ions (from the calcium acetate used for crystallization) tend to be nonspecifically bound on the surface (Fig. 4 ▶ *b*), with little or no anomalous signal.

The majority of the manually verifiable ions missed by our method were built as waters and flagged as heavier elements, but all candidate elements were rejected owing to poor agreement with the environmental criteria. In several cases where calcium was modeled in the original structures, the electron density was suggestive of a larger chemical entity (Fig. 4 ▶ *c*). Another example, PDB entry 3h50 (Axelrod *et al.*, 2010 ▶), contains a zinc coordinated by a glutamine OE1 atom, an uncommon interaction that was not used in our criteria (Zheng *et al.*, 2008 ▶). Although in these cases the other atomic properties, such as anomalous scattering, were consistent with the assigned element, other examples were identified that were clearly incorrect. For instance, the structure 2ii1 in the training set had four unidentified transition metals labeled as calcium, and 3obc (which was excluded from the tests) contained magnesium ions mistakenly built as zinc. In two cases the protocol identified genuine ions not present in the original structure, a Ca^2+^ site at a lattice contact in 3cjy (Fig. 4 ▶ *d*) and a Zn^2+^ site in 3h50 (Fig. 4 ▶ *e*).

As a further assessment of the ease of classifying ions, we also validated the deposited structures in our blind JCSG set using the *CheckMyMetal* server (Zheng *et al.*, 2013 ▶; http://csgid.org/csgid/metal_sites/), which uses some of the same analyses as our method but is limited to ions already modeled in the structure. The server flagged a large fraction of the originally built ions in the PDB as potentially problematic; only 43 calcium and 39 zinc ions passed all of the tests. This is attributable partially to our use of the less restrictive BVS and VECSUM criteria, and also the incorporation of anomalous data and other diffraction-based criteria. The majority of the flagged ions are nonetheless plausible based on visual inspection, but the validation results highlight the limits of solely relying on model features to unambiguously identify the element. Running the procedure with restrictive cutoffs for BVS and VECSUM reduced the success rate by approximately two-thirds (Table 1 ▶).

Finally, to assess the importance of anomalous data to our method, we ran the same blind test with Friedel pairs merged. Because most of the zinc ions were well ordered, the success rate was only slightly lower with merged data (Table 1 ▶). However, the results for calcium were significantly worse, with approximately a third of previously identified sites missed; for example, in PDB entry 3u7z the program placed and correctly identified seven out of 14 sites when *Phaser* was used but only two with merged data. This is because the criteria for coordinating Ca^2+^ are less stringent when the site has compatible anomalous scattering. Although many sites are recognizable as likely ions based on inappropriately close contacts with nearby O atoms, at partial occupancy the nonspecifically bound calciums cannot be reliably distinguished from sodium without an anomalous map peak or *Phaser* substructure site.

### 3.2. Other transition metals   

The same approach described above is applicable to structures containing other less common metals, which are expected to be found in their native environments in most cases. (We have not attempted to place iron–sulfur clusters or the central iron in heme rings, as these are best treated as special cases of ligand fitting.) However, the similar chemical and diffraction properties of the transition metals make it difficult to distinguish between a choice of elements if a mixture is expected or the identity is uncertain.

#### 3.2.1. *E. coli* YghZ (Ni^2+^)   

Although nickel occurs natively in some proteins, its use in affinity-tag purification (and some crystallization solutions) also leads to nonspecific binding. The structure 3n6q (Totir *et al.*, 2012 ▶) contains cations mediating crystal contacts at equivalent sites for the eight monomers in the asymmetric unit; although these were originally refined and deposited as magnesium, the *mF*_o_ − *DF*_c_ and anomalous difference maps indicate a much heavier element. Because the purification method used a nickel-bound chelating resin, this is the most likely candidate; following the removal of a spurious alternate conformation for an Arg side chain, *phenix.refine* was able to fit Ni^2+^ in all eight sites (Fig. 5 ▶ *a*).

#### 3.2.2. Carbonic anhydrase (Cd^2+^)   

The heavy metal cadmium is uncommon in native biological contexts, having only been observed as an essential cofactor in carbonic anhydrase from marine diatoms (Lane *et al.*, 2005 ▶; Xu *et al.*, 2008 ▶). However, because Cd^2+^ can substitute for Ca^2+^, it is an important toxin and may be used deliberately in crystallo­graphic studies. Additionally, its common use as an ingredient in commercially available crystallization buffers and additives, as well as the reasonable phasing power for SAD/SIR/MIR experiments, result in a relatively large number of cadmium-bound PDB structures (Fig. 1 ▶). In the carbonic anhydrase structures (PDB entries 3bob, 3boe and 3boh), a single cadmium is bound by each domain in approximately trigonal bipyramidal geometry by a pair of cysteines, one histidine and solvent atoms (Fig. 5 ▶ *b*). Owing to the accuracy of the valence calculations at high resolution, *phenix.refine* was able to identify the cadmium ions even with merged data. Addition of anomalous data enabled further identification of a chloride ion at one of the coordinating solvent sites in 3bob (Fig. 5 ▶ *b*).

### 3.3. Application to lighter cations   

We initially targeted the heavier cations, whose scattering profiles make them more easily recognized. However, given sufficiently high resolution, the method is equally valid for ions that are nearly isoelectronic with water (*e.g.* Na^+^ and Mg^2+^). In contrast to previous work (Nayal & Di Cera, 1996 ▶), we have found it difficult to reliably use valence calculations alone at more moderate resolution; the limit for this method has been suggested to be approximately 1.5 Å (Müller *et al.*, 2003 ▶). As for the heavier ions, the method is expected to work best for physiologically relevant sites with full occupancy and well ordered coordination shells (typically with octahedral geometry). The lack of significant anomalous scattering and similarity to water mean that the cutoffs for accepting a candidate must be much stricter, with a narrow range of permissible values for bond valence.

#### 3.3.1. Thrombin (Na^+^)   

The protease thrombin is known to bind sodium natively (Di Cera *et al.*, 1995 ▶) and has previously been used as a model system for ion identification based on valence calculations of solvent atoms (Nayal & Di Cera, 1996 ▶). We examined a set of ten ligand-bound structures (Biela *et al.*, 2012 ▶) determined at near-atomic resolution (between 1.27 and 1.90 Å), which we have used for testing automated ligand-placement and refinement (Echols *et al.*, 2014 ▶), Each of these has two sodium ions modeled in the deposited structure, one internal and one bound by crystal contacts, both present at full occupancy with excellent density and coordination shells. For these tests the process was started from the original molecular-replacement search model without ligands; the structures were solved and refined automatically to within 2% of the final *R*_free_ values. In eight of the structures (PDB entries 3p17, 3qto, 3qwc, 3sha, 3shc, 3si3, 3si4 and 3sv2) both sodium ions were placed and identified in the final round of refinement (Fig. 5 ▶); in 3qx5 a single ion was placed. The only false positive was a spurious extra ion identified in 3qtv.

#### 3.3.2. Protein kinase A (Mg^2+^)   

The cyclic AMP-dependent protein kinase (or PKA) was the first protein kinase to be crystallized (Knighton *et al.*, 1991 ▶) and both its regulation and enzymatic mechanism have been extensively studied. Like the majority of proteins in this family, it binds ATP in the central cleft, coordinated by two magnesium ions with approximately octahedral geometry. The AMP-PNP-bound structure 4dfx (Bastidas *et al.*, 2012 ▶) was selected as a test case since high-resolution (1.35 Å) data are available. The magnesium sites were clearly recognizable based on the geometry and the bond valences (2.09 and 2.19 *versus* a theoretical value of 2.0). The connectivity analysis of coordinating atoms was essential to identify both ions because one of the P atoms comes within less than 3.0 Å of one magnesium, although it does not directly coordinate it (Fig. 6 ▶). This structure also provides an example of an N atom (substituting for an O atom in AMP-PNP) coordinating magnesium, an interaction that is rare in both the PDB and the CSD (Zheng *et al.*, 2008 ▶; Harding, 1999 ▶, 2001 ▶).

## 4. Discussion   

To the best of our knowledge, our implementation of this protocol in *phenix.refine* is the first such example of the incorporation of automated ion placement into crystallo­graphic building and refinement. However, the method used draws upon a number of previous bioinformatics/chemo­informatics surveys and analytical tools cited in §[Sec sec1]1, and can also be thought of as an extension of automated solvent picking (Turk, 1992 ▶, 2013 ▶; Lamzin & Wilson, 1993 ▶; Afonine *et al.*, 2012 ▶) and anomalous substructure completion (Bricogne *et al.*, 1997 ▶; Read & McCoy, 2011 ▶). A comprehensive approach to model completion would of course encompass the detection of larger ions and other small molecules commonly found in crystallization solutions, such as ammonium, sulfate, phosphate or acetate, but these require a more sophisticated analysis of electron density.

We have chosen to focus on the simplest case here because it is more model-centric and easily integrated with existing building and refinement workflows. We have also concentrated on identification of the specific ion type because this directly improves the refinement model; this process is deliberately somewhat conservative, because assignment of ions is a scientifically important decision. It should also be informed by knowledge of the macromolecule and crystal conditions. Like any automated procedure, this implementation is only as reliable as the crystallographer responsible for validating and interpreting the results. Careful manual inspection and appropriately skeptical treatment of the output remains essential, especially for features that are likely to be functionally significant. In future, we intend to provide additional aids to manual completion by easy access to anomalous maps and *f*′ *versus* *f*′′ analysis, by listing individual diagnostics such as valence-bond parameters and short distances to specified ligand atom types, and perhaps for each isolated peak by estimating its likelihood of being a water, an alternate conformation, a positive ion or a negative ion.

As expected, the presence of residual difference map peaks and/or unusually low water *B* factors are nearly always diagnostic for heavier ions, and these features are most often used for manual placement. However, it is essential to also take the local chemical environment into account, especially when the identity of ions is not known with certainty. In the case of sodium and magnesium, only the coordination geometry and bond valence can distinguish these ions from water unless the data extend to true atomic resolution. (For this reason, it is likely that the number of sodium ions in the PDB is greatly underestimated.) The need for comprehensive analysis of both the environment and scattering properties becomes especially urgent when using automated solvent-placement routines, as these often build waters in density for missing protein residues or other small molecules. Although not explored in this work, consideration of the shape as well as height of density peaks may also be helpful in increasing the sensitivity without increasing false positives.

Although we have focused primarily on cations in this work, the identification of halides is also an essential extension of solvent placement. Because chloride is a ubiquitous component of purification and crystallization buffers, it is frequently seen in crystal structures (more than 6000 entries in the PDB as of February 2013), and our analysis of recently deposited structures suggest that it is in fact far more common but often unmodeled (N. Echols, unpublished work). This is in part because of the difficulty of distinguishing it from water owing to its relatively low electron count (often accompanied by partial occupancy) and nonspecific binding near the surface of the protein (Dauter & Dauter, 2001 ▶), and because anomalous data are not commonly used in refinement. Halide ions can be identified based on shorter than hydrogen-bonding distances to amide groups, especially the backbone N atom, and positively charged atoms. However, our tests have shown this to be significantly more difficult than the detection of cations at worse than atomic resolution (approximately 1.5 Å). In large part this is owing to the similarity of very well ordered waters and partial-occupancy chlorides when no significant anomalous signal is available. Although anecdotal evidence indicates that even at short wavelengths (near the Se edge, approximately 0.98 Å) this signal can sometimes be measured with sufficient accuracy to be detected by *Phaser*, this is frequently not the case.

### 4.1. ‘Corner cases’ and other common causes of failure   

Our testing identified several real-world scenarios that are especially problematic. As noted by Harding *et al.* (2010 ▶), it is extremely difficult to distinguish transition metals based on the model alone, and in practice we found that owing to the wide range of tolerances for the various element parameters, the results are inconclusive when there are multiple possible candidates. If a metal ion is known or suspected to bind but the identity is ambiguous, additional biochemical or bio­physical data are required; with suitable X-ray sources and beamlines, this might include multi-wavelength diffraction or X-ray spectroscopy, which are commonly used as part of the JCSG structure-determination process. One potential future extension of our method is to incorporate analysis of MAD data sets in the refinement procedure. An extreme case is when the sites have mixed occupancy; an example from the JCSG training set is PDB entry 3qxb, which contains multiple sites experimentally confirmed to be a mixture of Mn^2+^ and Fe^2+^. The halides are problematic mostly owing to their ability to bind nonspecifically; as a result, only the most common interactions (such as binding to amine groups or cations) are currently used. A more physically realistic energy function could be useful to evaluate the favorability of the local environment more accurately (*e.g.* Zhang *et al.*, 2012 ▶).

Like many automated building methods, our protocol, although effective with good data (and accurate models), is limited by map quality, and loses sensitivity at low resolution. This is in part owing to the limited information available in the difference maps, which may be overcome by a greater reliance on the anomalous difference map and identification of common binding sites such as tetrahedral metal centers and nucleotide phosphates. The lighter cations sodium and magnesium present further difficulties, especially as their coordination shells are not easily resolved when atoms do not form clearly defined peaks of electron density, and the lack of anomalous scattering makes more approximate rules un­feasible. For magnesium, a more direct approach to identify octahedral geometries may be required (see, for example, Klein *et al.*, 2004 ▶). Other common obstacles include the following.

(i) Partial occupancy, which typically makes the use of physical atomic properties less reliable, as both the real scattering and the refined *f*′′ are reduced. This is problematic for structures crystallized in high concentrations of heavier elements, and even more so for chloride ions.
(ii) In some cases the refined *f*′′ exceeds the cutoff for the expected element; this may be owing to inherent inaccuracies in the anomalous data.
(iii) Although some attempt has been made to account for static disorder in the model when assessing coordination shells, it is not currently attempted for the ions. This may result in sites being rejected owing to a correlated alternate conformation of a protein side chain, or because they lie too close to a previously identified ion (*i.e.* a split site).
(iv) In some cases the orientation of Asn, Gln and His side chains may be flipped in the incomplete structure, resulting in unfavorable interactions for potential cations. Although these side chains are optimized during refinement using *Reduce *(Word *et al.*, 1999 ▶), this is performed in the context of existing water molecules (if any), without knowledge of the ion binding site. Additionally, the ambiguity of histidine proton­ation states in partial models may prevent initial water placement if explicit H atoms are built. In future, the ion analysis and the *Reduce* flip and H-atom assignment should be integrated in order to take into account the joint effect of their choices.
(v) Even seemingly minor errors in model geometry may prove limiting if the model is further distorted during refinement to compensate for absent heavy atoms. In the YghZ/3n6q example (Totir *et al.*, 2012 ▶), building of the nickel ions required an initial geometry-minimization step to prevent coordinating carboxyl groups from being pulled into nickel density. Similar errors have been observed in published structures (*e.g.* Rimsa *et al.*, 2011 ▶). Especially at less than atomic resolution, refinement as water will push apart an ion and its ligands somewhat, making the valence-bond analysis less reliable.
(vi) Less common, but equally vexing from the perspective of automation, are unusual chemical interactions that are excluded by the initial filtering step. In rare cases, at basic pH it may be possible for positively charged amide groups such as N-termini or Lys N^∊^ to become deprotonated and interact with metal cations. However, because the proximity of these groups would nearly always indicate an unfavorable environment, such ions are missed by our method.

Finally, we emphasize that the use of anomalous data is an especially powerful criterion for accurately identifying many bound ions (Mueller-Dieckmann *et al.*, 2007 ▶; Thorn & Sheldrick, 2011 ▶). For this reason, even when experimental phasing is unnecessary and/or no significant anomalous scattering is expected from the structure(s) being crystallized, researchers may benefit from collecting complete anomalous pairs and using these in refinement. If crystals are sufficiently plentiful and/or radiation-tolerant, it may also be worthwhile collecting an additional lower-resolution data set at a longer wavelength. Furthermore, we strongly encourage the deposition of the separate Friedel pairs (or, better yet, the unmerged intensities) in the Protein Data Bank upon publication, as these provide essential information about the experiment and may lead to future improvements in both the development of crystallographic software and in the deposited structures themselves (Joosten *et al.*, 2009 ▶, 2012 ▶; Rimsa *et al.*, 2011 ▶; Afonine *et al.*, 2012 ▶).

## 5. Availability   

The method as implemented in *phenix.refine* is available as part of *PHENIX* version 1.8.4 or more recent, which is free of charge for academics (http://phenix-online.org/). Source code is included in the distribution; most of the core analysis code (including parameter sets) is available under an open-source license as part of *cctbx* (http://cctbx.sf.net).

## Supplementary Material

Criteria for ion assignment, listing of structures in benchmark set, and figures showing distribution of ion occupancies and B factors.. DOI: 10.1107/S1399004714001308/lv5059sup1.pdf

## Figures and Tables

**Table 1:** Statistics for blind test on JCSG structures containing Ca^2+^ and/or Zn^2+^, indicating the number of each ion type successfully placed and identified by *phenix.refine* and in agreement with the deposited model Numbers in parentheses indicate false positives and genuine ions not present in the original structures.

	Ca^2+^	Zn^2+^
In PDB (without alternates)	121	98
Built with default settings, no H atoms	46 (2, 1)	77 (0, 1)
Built with default settings, explicit H atoms	48 (1, 1)	75 (0, 1)
Built without anomalous data	36 (2, 0)	73 (2, 0)
Built with valence required	17	25

## Footnotes

1 Supporting information has been deposited in the IUCr electronic archive (Reference: LV5059).
