# Supplementary material for: Automated identification of elemental ions in macromolecular crystal structures
Source: Acta Crystallogr D Biol Crystallogr. 2014 Mar 20;70(Pt 4):1104–14. doi: 10.1107/S1399004714001308 (PMC3975891; doi:10.1107/S1399004714001308)

Acta Crystallographica Section D

Volume 70 (2014)

Supporting information for article:

Automated identification of elemental ions in macromolecular crystal structures

Nathaniel Echols, Nader Morshed, Pavel V. Afonine, Airlie J. McCoy, Mitchell D. Miller, Randy J. Read, Jane S. Richardson, Thomas C. Terwilliger and Paul D. Adams

Table S1 Parameters and default tolerances for cation identification. These are also available in machine-readable form in CCTBX in the file mmtbx/ions/ion_parameters.cif. Tables S1 and S2 were based in part on Rulísek & Vondrásek 1998; Harding 2000, 2001; Muller et al. 2003; Dokmanic et al. 2008; Zheng et al. 2008.

Ion	VECSUM (max)	# of coordinating atoms	Minimum # of non-water coordinating atoms	Bond valence sum (upper and lower bounds)
Na^+	0.4	4-8	1	0.8-1.2
Mg^{2+}	0.4	4-6	0	1.8-2.2
K^+	0.4	4-8	1	0.8-1.2
Ca^{2+}	0.4	5-8	0	1.75-2.2
Mn^{2+}	0.4	4-6	1	1.75-2.2
Fe^{2+}	0.4	5-6	1	1.75-2.2
Co^{2+}	0.4	5-6	1	1.75-2.2
Cu^{2+}	0.4	3-4	1	1.75-2.2
Ni^{2+}	0.4	4-6	1	1.75-2.2
Zn^{2+}	0.4	4-6	1	1.75-2.4
Cd^{2+}	0.6	3-7	1	1.5-2.3

Table S2 Additional filtering criteria for cations.

Ion	Coordinating atoms	Coordinating protein sidechains	Geometry	Coordinating protein backbone atoms
Na^{+}	O	Any with oxygen atoms	Octahedral, square-pyramidal, none	O, OXT
Mg^{2+}	O, N	Ser, Thr, Asp, Asn, Glu, Gln, His	octahedral	O, OXT
K^{+}	O	Any with oxygen atoms	any	O, OXT
Ca^{2+}	O	Any with oxygen atoms	Octahedral, pentagonal-bipyramidal, none	O, OXT
Mn^{2+}	O, N	Ser, Thr, Asp, Asn, Glu, Gln, His	any	-
Fe^{2+}	O, N, S	Cys, Asp, Glu, His, Tyr	any	-
Co^{2+}	O, N	Cys, Asp, Glu, His	Tetrahedral, octahedral	-
Cu^{2+}	O, N, S	Cys, Met, His	Tetrahedral, square planar, none	-
Ni^{2+}	O, N, S	Cys, Asp, Glu, His	Tetrahedral, octahedral, None	-
Zn^{2+}	O, N, S	Cys, Asp, Glu, His	Tetrahedral, octahedral, none	-
Cd^{2+}	O, N, S	No restrictions	Tetrahedral, octahedral, none	O, OXT

Table S3 Individual structures in the JCSG blind set.

PDB ID	Resolution (Å)	Ions	Notes
2fm1	2.25	2 CA	
2gf6	1.91	1 CA	
2gvi	1.87	5 ZN	(Axelrod, Das et al. 2010)
2qpx	1.40	2 ZN	
3cjy	1.70	1 CA	
3db7	1.40	3 CA	
3dzz	1.61	2 CA	
3fd0	2.12	1 CA	
3g0k	1.30	1 CA	
3h50	1.60	4 ZN	(Axelrod, Kozbial et al. 2010)
3jtx	1.91	1 CA	
3ju7	2.19	4 CA	
3llx	1.50	1 ZN	
3lub	12.11	12 CA, 24 ZN	
3luu	1.93	1 ZN	
3lwu	2.10	1 ZN	
3m83	2.12	8 CA	(Levisson et al. 2012)
3mbj	2.10	3 ZN	
3mcx	1.49	6 ZN	
3mdo	1.91	7 CA	
3mzo	1.98	11 CA	1 alt. conf.
3n91	2.40	1 CA	
3na6	2.00	1 CA	
3nqn	1.88	3 CA	
3o14	1.70	2 ZN	
3ohe	1.20	3 CA	1 alt. conf.
3oqq	2.08	4 CA	
3orj	2.16	1 ZN	
3oru	1.11	1 ZN	
3p1v	1.93	4 ZN	

3pfo	1.90	4 ZN	two of these appear to be something lighter (no anomalous)
3pgv	2.39	4 CA	
3pw3	2.23	6 ZN	
3qc0	1.45	1 ZN	annotated as partial (or mixed) occupancy
3qov	2.20	4 ZN	
3qta	2.00	3 CA	
3r4i	2.24	13 CA	
3s6f	1.19	2 CA	
3son	1.71	2 CA	
3tc8	1.06	2 ZN	
3u1w	2.00	4 CA	
3u7z	1.30	14 CA	2 alt. confs.
3urz	2.19	4 ZN	
4dgu	2.37	9 ZN	
4e5v	1.75	2 ZN	
4e6r	2.20	5 ZN	
4ecg	2.30	9 CA	
4epz	1.68	2 CA	
4esn	2.20	2 ZN	
4f53	2.25	3 CA	
4iej	1.30	1 CA	
4fuu	1.45	1 ZN	
4leh	2.90	1 ZN	
4lqx	2.34	2 ZN	

Figure S1 Histograms of refined occupancies for (A) calcium ions and (B) zinc ions in the JCSG "blind" test set. Purple bars represent statistics for the deposited structures re-refined in *phenix.refine*; green bars represent statistics only for the ions that could be identified correctly. X-axes (but not y-axes) are on the same scale in both plots.

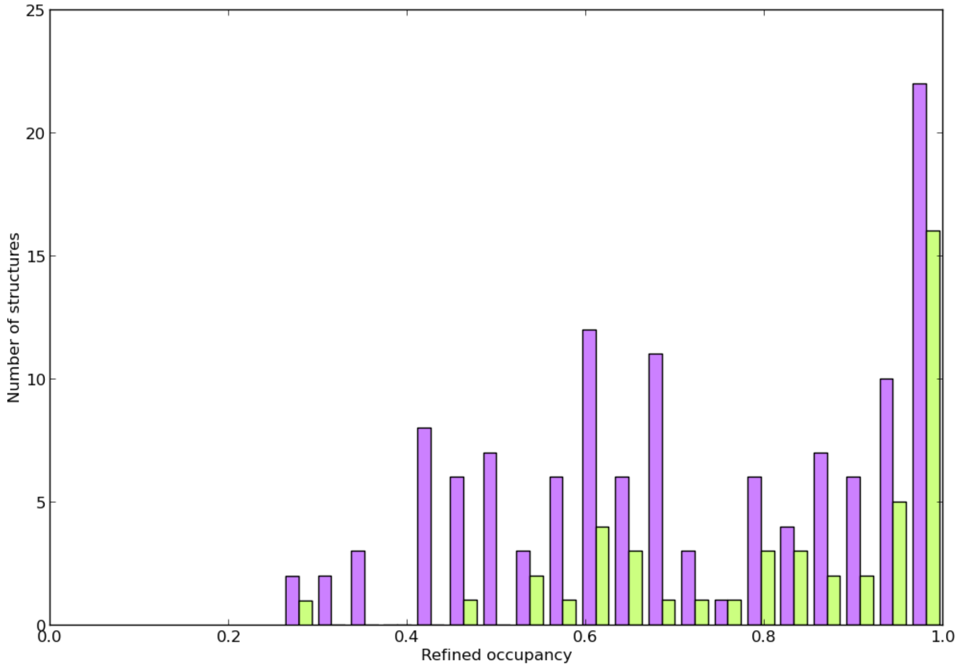

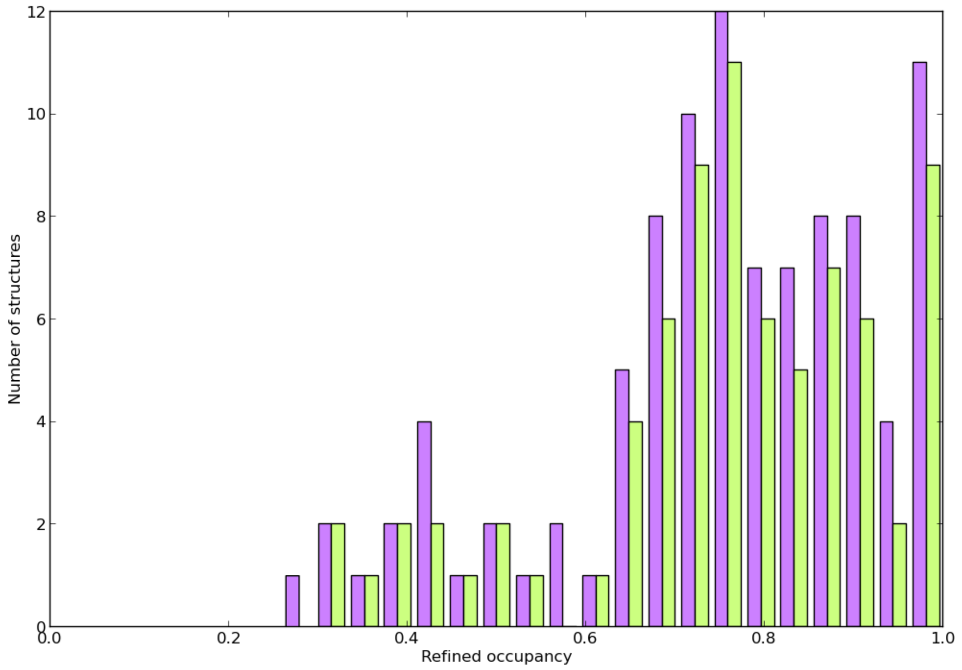

Figure S2 Histograms of normalized refined total isotropic equivalent B-factors for (A) calcium ions and (B) zinc ions in the blind test set, calculated by dividing the ion B-factor by the mean for all non-hydrogen atoms in a given structure. Colors are as in Figure S1.

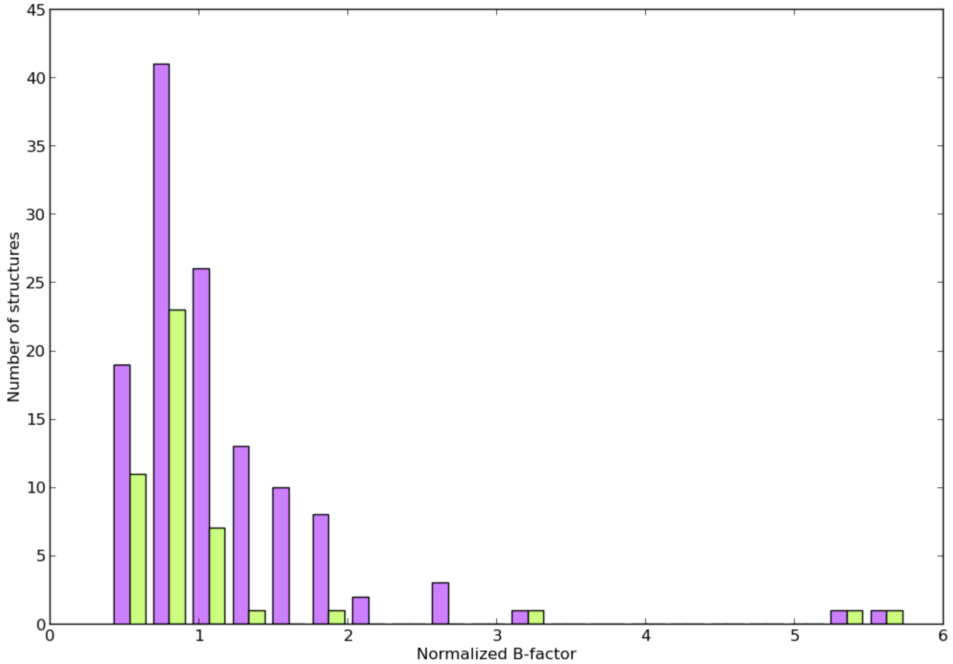

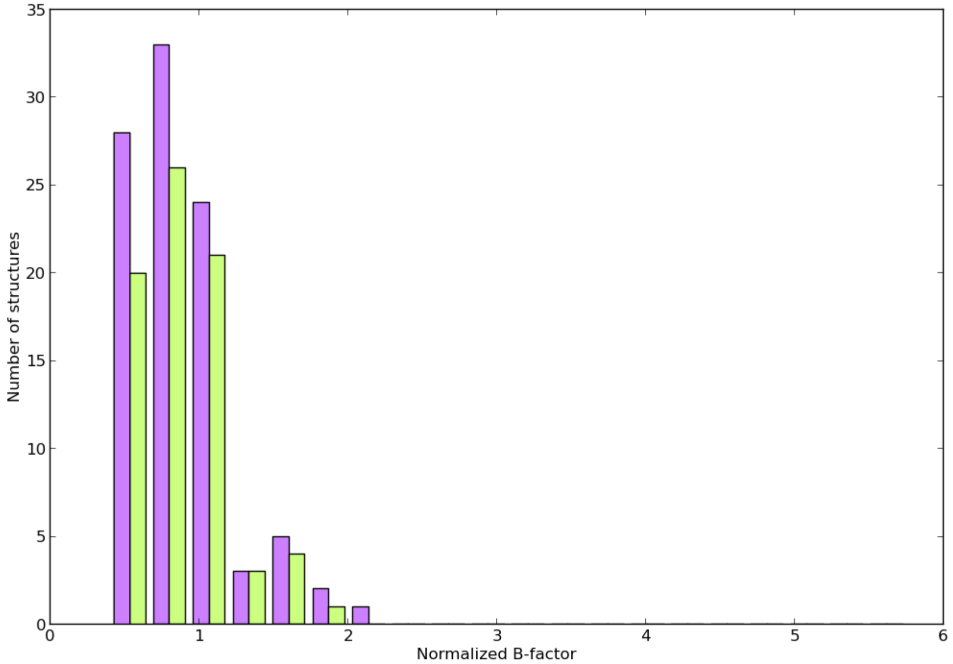

Supplement: Supplementary file 1 [file d-70-01104-sup1.pdf]
